# Effects of 45S5 bioactive glass on the remineralization of early carious lesions in deciduous teeth: an in vitro study

**DOI:** 10.1186/s12903-021-01931-3

**Published:** 2021-11-12

**Authors:** Rui Zhang, Jianyan Qi, Min Gong, Qian liu, Hongyan Zhou, Jue Wang, Yufeng Mei

**Affiliations:** 1 Department of Pediatric Dentistry, The Affiliated Stomatological Hospital of Nanjing Medical University, Shanghai Road 1st, Nanjing, 210029 China; 2 Jiangsu Key Laboratory of Oral Diseases, Nanjing, China

**Keywords:** Bioactive glass, Remineralization, Deciduous teeth, Energy dispersive X-ray spectroscopy, Fourier transform infrared spectroscopy equipped with attenuated total reflectance

## Abstract

**Background:**

Early childhood caries has been designated as a serious public health problem. The traditional restoration method is very challenging, especially in uncooperative patients. Non-invasive therapy, like remineralization agents, which have been developed to reverse the demineralization progress at the early stage of caries, may be a better choice. This study aimed to evaluate the remineralization efficacy of different concentrations of 45S5 bioactive glass (BAG) on artifical carious lesions of deciduous enamel.

**Methods:**

65 caries-like enamel lesions of the deciduous teeth were assigned to 5 groups (n = 13) and transported to a 14 days pH-cycling: Group A: 2%BAG, Group B: 4%BAG, Group C: 6%BAG, Group D: 8%BAG, and Group E: deionized water (DDW, negative control). 8 sound (Group F) and 8 demineralized teeth (Group G) were prepared for contrast. The recovery power of mechanical property was evaluated by Vickers hardness test through the recovery of enamel microhardness (%REMH). Surface morphology, mass fraction of Ca and P ions, and Ca/P atomic ratio were analyzed by scanning electron microscopy coupled with energy-dispersive X-ray spectroscopy (EDX). Moreover, Fourier transform infrared spectroscopy equipped with attenuated total reflectance was used to identify the chemical structure of newly formed compounds.

**Results:**

% REMH were (42.65 ± 1.35), (52.59 ± 2.96), (57.40 ± 1.72), (52.91 ± 2.55), (12.46 ± 2.81) in 2%BAG, 4%BAG, 6%BAG, 8%BAG, and DDW groups respectively. Micro-spherical particles were deposited in all BAG groups and 6% BAG showed the densest and most uniform surface. EDX analysis identified significantly higher Ca(wt%) and P(wt%) in four BAG groups than in the demineralized group (*p* < 0.005), while 6% BAG showed the highest mineral gain efficacy. The infrared spectrum demonstrated that newly mineralized crystals were consisted of type-B hydroxycarbonate apatite.

**Conclusion:**

BAG possessed a promising remineralization effect on artificial lesions in deciduous enamel by recovering enamel surface mechanical property, morphology and chemical elements. Among them, 6% BAG performed the greatest overall efficacy. Acting as a new caries-arresting biomaterial, 45S5 BAG has the potential to facilitate the adaptation of better carious prevention strategies in children.

## Introduction

Early childhood caries is a chronic infectious disease affecting 30–60% of preschool children across the globe [1], and has been considered a serious public health problem. It spreads very fast and may cause severe pain, swelling, facial aesthetic problems [2] and abnormal pronunciation in children under 71 months [1, 3]. Caries form when the balance of demineralization and remineralization is disturbed, resulting in changes in the morphology and chemical structure of the enamel. The traditional restoration method to treat deciduous tooth caries is full of challenges, especially in uncooperative patients. Non-invasive therapy to reverse the demineralization progress at the early stages of caries may be a better choice. Many mineralization agents have emerged these years to maintain enamel integrity and prevent the occurrence of carious cavities.

In recent years, BAG has been introduced into many fields of dentistry due to its high biocompatibility [4, 5]. It exerts remineralization effects on both enamel [6] and dentin [7] and has low cytotoxicity for dental pulp cells [8]. In addition, its antimicrobial activity against intraoral bacteria has also been confirmed [9]. BAG is a multi-component inorganic compound composed of sodium, calcium, phosphorus, and silica (sodium-calcium phosphosilicate) [10]. It showed the capabilities of acid-neutralization and absorbing calcium (Ca) ions via its functional groups to form HCA in physiological conditions [5]. It is a promising agent for remineralization owing to its ability to act as a biomimetic mineralizer.

To the best of our knowledge, studies on the remineralization of BAG mainly focused on the dentin and permanent enamel. Wu et al. [10] proved that BAG paste could improve the microhardness and promote mineral deposition on the superficial layer of the demineralized dentin. Abbassy et al. [11] found that BAG paste successfully reduced the lesion depth and mineral loss of permanent enamel. However, there is still a lack of evidence on its exact efficacy on deciduous teeth. Besides, the paste [10–12] and the commercial BAG-containing toothpaste [13, 14] used in existing studies are full of additives, whose role is not clear. Based on these, we designed a study to evaluate its independent role on remineralization in deciduous enamel with a pure suspension form to reduce interference by any additive.

## Material and methods

### Sample size calculation

The sample size calculation was performed using G*Power 3.1. Based on a previous study in remineralization regarding microhardness and mineral content [15], we estimated effect size of 0.8 with α = 0.05/β = 0.2; the total sample size computed was 25 for 5 groups for microhardness test and 35 for 7 groups for EDX test. For qualitatively analyzing the chemical structure by FT-IR/ATR, 3 samples of all of the 7 groups were needed. Thus, the total sample size was determined as 81.

### Specimen preparation

The experiment was designed as an in vitro one and was approved by the Ethics Committee of The Affiliated Stomatological Hospital of Nanjing Medical University (approval number PJ2021-004-01). Deciduous incisors identified as "deciduous tooth retention" requiring extraction were collected if there were no signs of a cavity, stains, crack, abrasion, or hypoplasia. A high-speed diamond disc removed the tooth root. The remaining crown was embedded in epoxy resin, leaving only the facial surface exposed and polished by silicon carbide paper (800, 1200, 2000 grit size, China) to achieve a flat enamel surface. In the next stage, a window of 3 × 3 mm^2^ was outlined on the equatorial area, and the rest of the tooth surface was covered by water-resistant nail varnish three times. The enamel blocks were then subject to sonication for 5 min and rinsed with DDW, and stored in 0.4% thymol solution until further processing.

### Baseline microhardness evaluation

Five samples from each experimental group were randomly selected for microhardness evaluation. A Vickers hardness testing machine with a load of 100 g with a dwell time of 10 s measured the hardness of five points, at least 100 µm apart from each other in the polishing enamel area. The average value of 5 indentations was calculated as Vickers Hardness Number 0 (VHN_0_) of each sample.

### Preparation of de-/remineralizing solution

Demineralizing and remineralizing solutions were made up of analytical-grade chemicals and distilled water. The demineralizing solution comprised 0.05 M acetic acid, 2.2 mM KH_2_PO_4_, and 2.2 mM CaCl_2_ with pH adjusted to 4.5 using KOH. The remineralizing solution which contained 1.5 mM CaCl_2_, 0.9 mM NaH_2_PO_4_, and 0.15 M KCl had a pH of 7.0 [13]. The pH adjustment was evaluated using a pH electrode calibrated to three solutions of known pH, 4.01, 7.0, and 10.01.

### Artificial carious lesion creation

Except for the eight sound enamel blocks, all the remaining 73 samples were immersed in demineralization solution (10 mL/sample) and soaked at 37 °C for 48 h to create an artificial incipient caries-like lesion. The solution was freshly prepared every day and was changed at a 24 h interval. The samples were rinsed with DDW for 3 min followed by ultrasonic cleaning in DDW 5 min to terminate demineralization. Specimens were then stored in a refrigerator at 4 °C with a relative humidity of approximately 100% for subsequent experiments.

### Evaluation of microhardness after demineralization

Post-formation of caries-like lesions, samples from each group that had previously participated in the hardness test in the experimental group were tested again with the same method to obtain the respective values after demineralization. The average value of each sample was defined as Vickers Hardness number 1 (VHN_1_).

### Group assignment

**Fig. 1:**
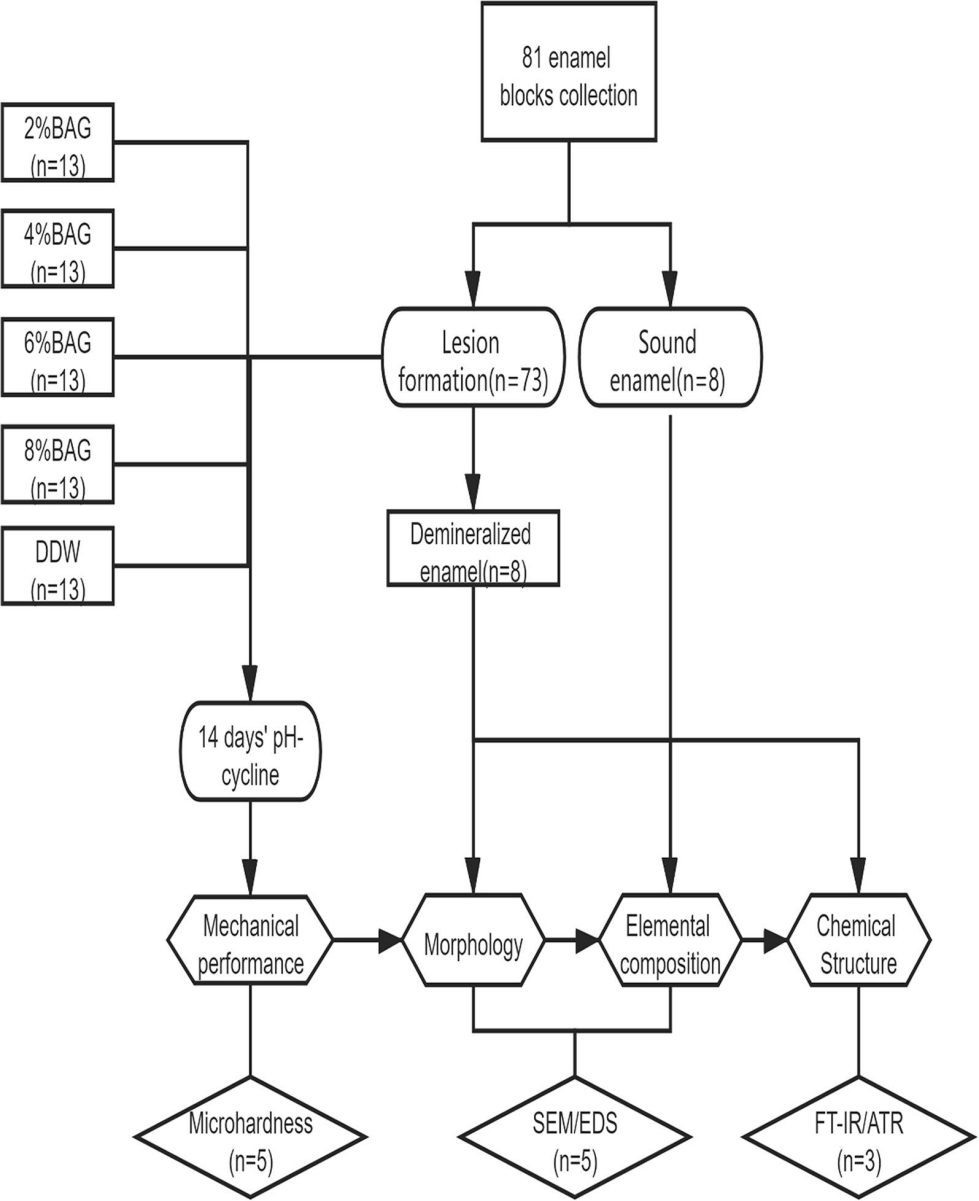
Flow chart of the experiment schedule

Sixty-five samples were randomly assigned to five groups (n = 13) for pH-cycling. The remaining sixteen blocks were not subjected to the pH-cycling treatment and were served as two control groups (n = 8) (Fig. 1).

- Group A: 2% BAG suspension (Mix 0.2 g 45S5 BAG nanoparticles to 9.8 g DDW)
- Group B: 4% BAG suspension(Mix 0.4 g 45S5 BAG nanoparticles to 9.6 g DDW)
- Group C: 6% BAG suspension(Mix 0.6 g 45S5 BAG nanoparticles to 9.4 g DDW)
- Group D: 8% BAG suspension(Mix 0.8 g 45S5 BAG nanoparticles to 9.2 g DDW)
- Group E: DDW-negative control
- Group F: Sound enamel
- Group G: Demineralized enamel

45S5 BAG nanoparticles were acquired by Datsing Bio-Tech Co. Ltd., Beijing, China. All the suspensions were kept under agitation before the brushing phase to maintain good suspension stability [16].

### The pH-cycling regime

A clinically relevant pH-cycling regime was conducted for 14 days to evaluate the remineralizing efficacy of the BAG. The pH-cycling procedure was implemented twice daily (5 min per application) at 08:00 am and 18:00 pm for 14 consecutive days. Each sample was first immersed in the demineralizing solution (5 mL per sample) for 10 min, followed by thorough rinsing with distilled water. Later, the samples were treated with respective remineralization agents for 5 min. In BAG groups, agents were continuously brushed onto the enamel window using a microbrush. In contrast, enamel surfaces were immersed in the DDW (5 mL/sample) for 5 min in the NC group. After that, they were rinsed under running DDW to remove the residual solution and incubated in artificial saliva solution, which was refreshed every day at 37 °C to simulate natural remineralization in an oral environment. After 14 days of the pH-cycling regime, samples were thoroughly rinsed under running DDW followed by ultrasonically cleaning in DDW water for 5 min to remove any residual agent.

### Post-treatment analyses

#### Surface microhardness

After the pH-cycling regime, samples from each group that had previously participated in the hardness test were tested again to obtain the respective values after remineralization. The average value of each sample was defined as Vickers Hardness Number 2 (VHN_2_). The percentage of recovery of enamel microhardness (%REMH) was defined as follows: % REMH = (VHN_2_ − VHN_1_)/(VHN_0_-VHN_1_)*100% [10].

#### SEM with EDX

After remineralization, five samples from each group were selected and dehydrated in an ascending ethanol series (50–100%). They were then gold coated. Surface morphologies were observed using a scanning electron microscope (Gemini 500, Carl Zeiss, Germany) operating at 20 kV in the secondary electron mode. EDX examined the surface chemical composition. Three random areas on each enamel window were chosen to identify Ca and P weight mass fraction and Ca/P atomic ratio.

#### FT-IR/ATR

FTIR spectrophotometer (Bruker, Germany) equipped with an ATR accessory(a monolithic diamond) was used to monitor the changes in the chemical structure of the enamel. Three samples from each group were selected and dehydrated in an ascending ethanol series (50–100%), and were placed on the diamond crystal top-plate of the ATR accessory with the exposed surfaces facing up. Absorbance spectra of each sample were collected with 4.0 cm^−1^ resolution in the wavenumber range of 1700–700 cm^−1^. Three spots were randomly chosen on the middle surface of each specimen. For mean spectrum calculation, the spectra from each group were baseline corrected. The spectral bands were analysed in the software Origin 2019b(HP Inc, USA) in order to assign the corresponding functional groups.

### Statistical analyses

The data obtained were analyzed in SPSS 25 for Windows (IBM, USA). One-way ANOVA with post-hoc Tukey’s test was used to analyze statistical differences among the microhardness values and %REMH. Data corresponding to weight percentages of Ca and P and Ca/P ratios fulfilled normality criteria (*p* > 0.05) but failed in the homogeneity of variance; thus Welch's analysis of variance (ANOVA) with Games-Howell post-hoc comparisons were used. The level of statistical significance was set at α = 0.05.

## Results

Table 1 demonstrates the microhardness values and % REMH obtained from each group. One-way ANOVA showed no significant difference in microhardness between groups at baseline (*p* = 0.917) or after 48 h demineralization (*p* = 0.923). After pH-cycling, the microhardness values of the BAG group were significantly higher than those of the DDW group and the 6% BAG group showed the highest. The %REMH demonstrates that all groups had re-hardened the enamel but differed significantly between groups (*p* < 0.001). The 6% BAG exhibited the highest surface microhardness recovery (57.40% ± 1.72) and was statistically higher than the other groups. No significant difference (*p* = 1.000) was found between 8% BAG (52.91% ± 2.55) and 4% BAG (52.59% ± 2.96). All of the BAG groups, including 2% concentration, had a significantly higher % REMH than the NC group (12.46% ± 2.81).

**Table 1:** Microhardness analysis of enamel followed by %REMH according to the treatments (means and standard error)

Groups	VHN_0_	VHN_1_	VHN_2_	%REMH
2%BAG	345.39 ± 7.43^A^	139.75 ± 8.29^B^	227.43 ± 4.41^Ca^	42.65 ± 1.35^a^
4%BAG	340.45 ± 15.62^A^	138.29 ± 6.14^B^	244.22 ± 4.47^Cb^	52.59 ± 2.96^b^
6%BAG	343.79 ± 10.15^A^	138.14 ± 5.40^B^	256.02 ± 3.91^Cc^	57.40 ± 1.72^c^
8%BAG	345.84 ± 6.97^A^	136.94 ± 1.44^B^	247.34 ± 4.51^Cb^	52.91 ± 2.55^b^
DDW(NC)	343.81 ± 5.56^A^	140.35 ± 8.40^B^	165.85 ± 3.64^Cd^	12.46 ± 2.81^d^

Distinct superscript letters indicate statistical significance among the treatments

*BAG* bioactive glass, *VHN*_*0*_ Vickers microhardness at baseline, *VHN*_*1*_ Vicker’s microhardness after demineralization, *VHN*_*2*_ Vickers microhardness after remineralization, *%REMH* recovery of enamel microhardness

**Fig. 2:**
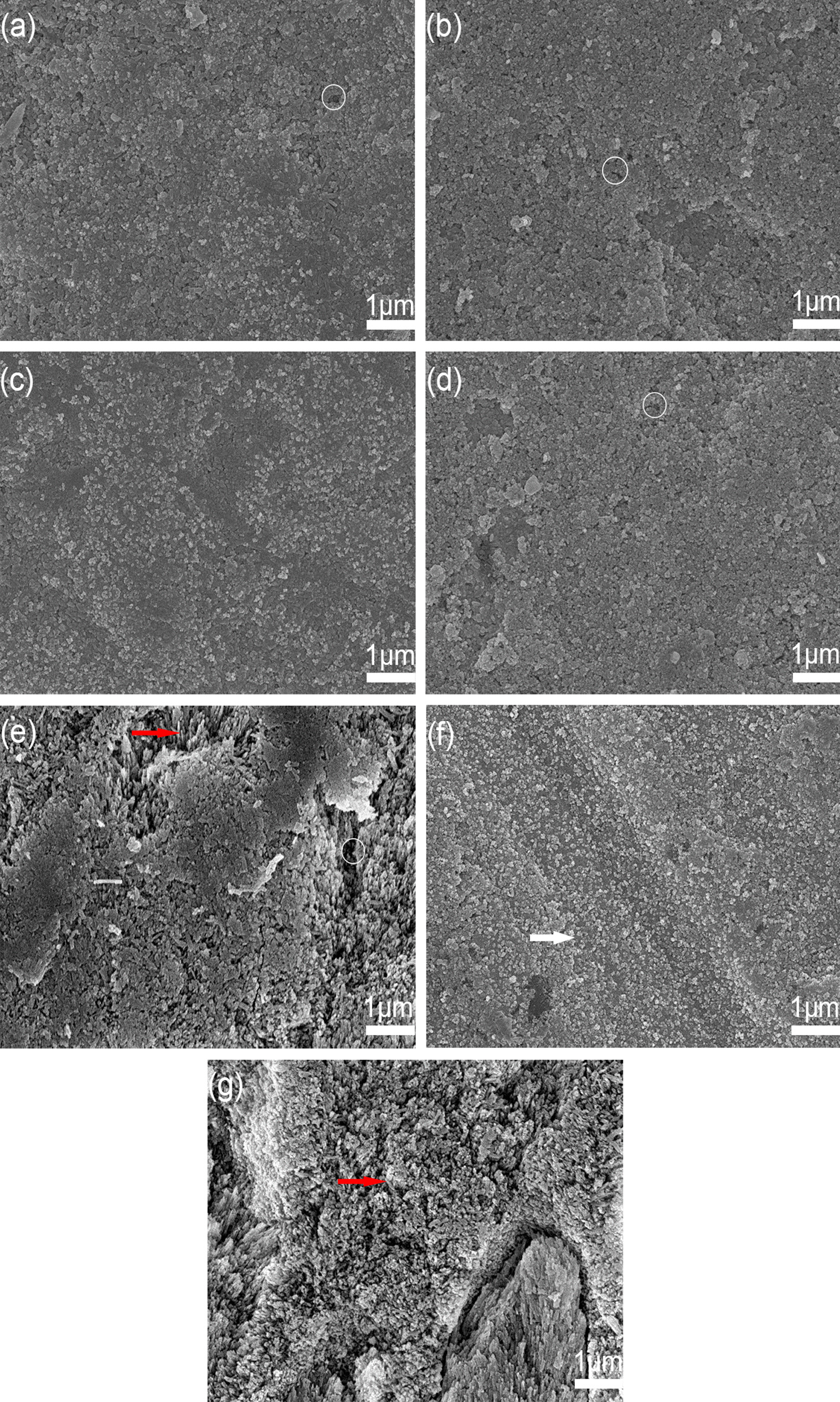
SEM Images of enamel surface in each group. Images with 2%, 4%, 6%, 8%BAG groups (**a**–**d**), DDW (**e**), sound enamel (**f**), demineralized enamel (**g**) at 10,000 × magnification. All BAG groups **a**–**d** showed a precipitation layer covering, while 6% BAG showed the most dense and integrated surface. Microporosities could be seen (white circle) in 2%BAG (**a**), 4%BAG (**b**), 8%BAG (**d**) group. Exposed enamel prisms (red arrows) could be seen in DDW (**e**) with a large number of pores (white circle). Thin and partial sediments found in (**e**) could be due to the re-deposition of dissolved Ca and P ions caused by remineralization solution. Regarding sound enamel (**f**), obvious scratches (white arrow) induced by polishing can be seen. After demineralization, abundant exposed enamel prisms with keyhole patterns could be seen (**g**), and no deposits were formed

Figure 2a–d showed the surface morphology of enamel in BAG groups after pH-cycling. Abundant micro-spherical particles were deposited in enamel voids and connected to cover the entire enamel surface. Sediments in different BAG groups were similar but slightly different in the number of exposed microporosities. Among them, the 6% BAG showed the densest and integrated surface deposition without noticeable pores, while 4% and 2% BAG groups had slightly more microporosities. In the DDW group, sediments were formed partially, which were thin and unevenly distributed. Figure 2f showed sound enamel morphology where polishing marks were noticeable. After demineralization, a large number of exposed enamel prisms with keyhole patterns could be seen (Fig. 2g), suggesting great mineral loss.

**Fig. 3:**
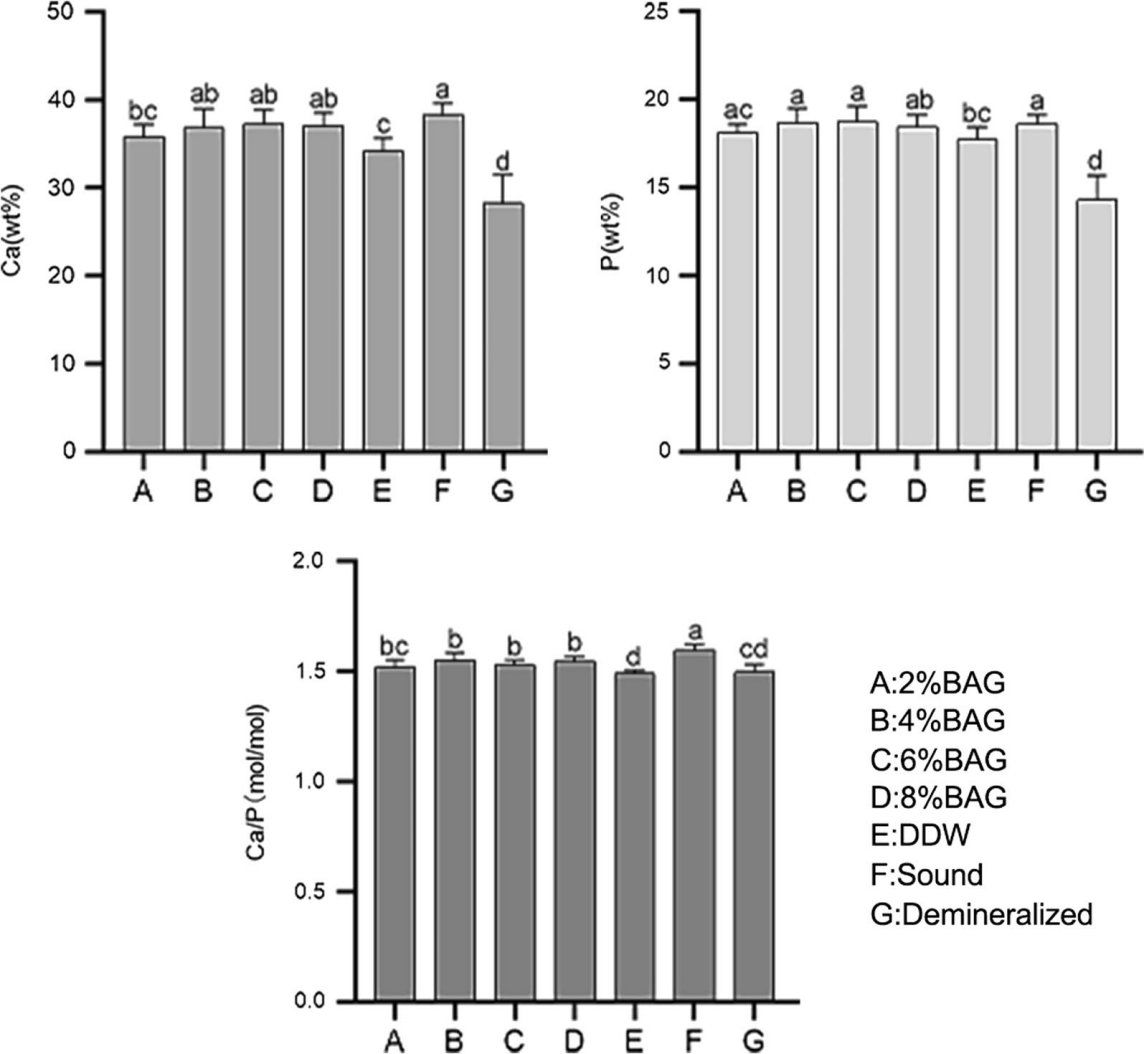
EDX analysis of weight percentage of calcium (Ca), phosphorous (P) and Ca/P (mol/mol) of each group. Lowercase letters indicate statistically significant differences

EDX analysis was used to quantitatively analyze the samples’ calcium and phosphorus contents (Fig. 3). Welch’s-ANOVA indicated that Ca(wt%) and P(wt%) were significantly higher in four BAG groups than those in the demineralized group. Furthermore, 4%BAG, 6%BAG and 8%BAG exhibited equal weight percentage of Ca to the sound enamel; among them, 6%BAG aquired the most Ca ions onto the demineralizaed surface. As to P ion, no significant difference was found between each BAG group and sound ones. Ca/P atonic ratio for the sound enamel was (1.60 ± 0.03) and decreased to (1.50 ± 0.03) after 48 h demineralization. After remineralization, the ratio increased in BAG groups, of which the 4% BAG (1.55 ± 0.03) and 8% BAG (1.55 ± 0.02) were the highest, followed by 6% BAG (1.53 ± 0.02). However, the difference between the BAG groups was not significant.

In sound enamel, the strongest bands were found at 985 cm^–1^ and 950 cm^–1^ which may be attributed to the v_3_PO_4_^3−^ and the v_1_PO_4_^3−^ vibration, respectively. The two peaks were shifted to higher wavenumbers (1008 cm^–1^and 956 cm^–1^) after demineralization and shift back after pH cycling in all the experimental groups except for the DDW group. The band at 869 cm^–1^ is attributed to v_2_ CO_3_^2–^(β-HCA), v_3_ CO_3_^2–^ presented bands at 1410 cm^–1^ and 1452 cm^–1^. No band at 880 cm^–1^ and 1545 cm^–1^ was seen in BAG groups, indicating that no type-A HCA was found.

**Fig. 4:**
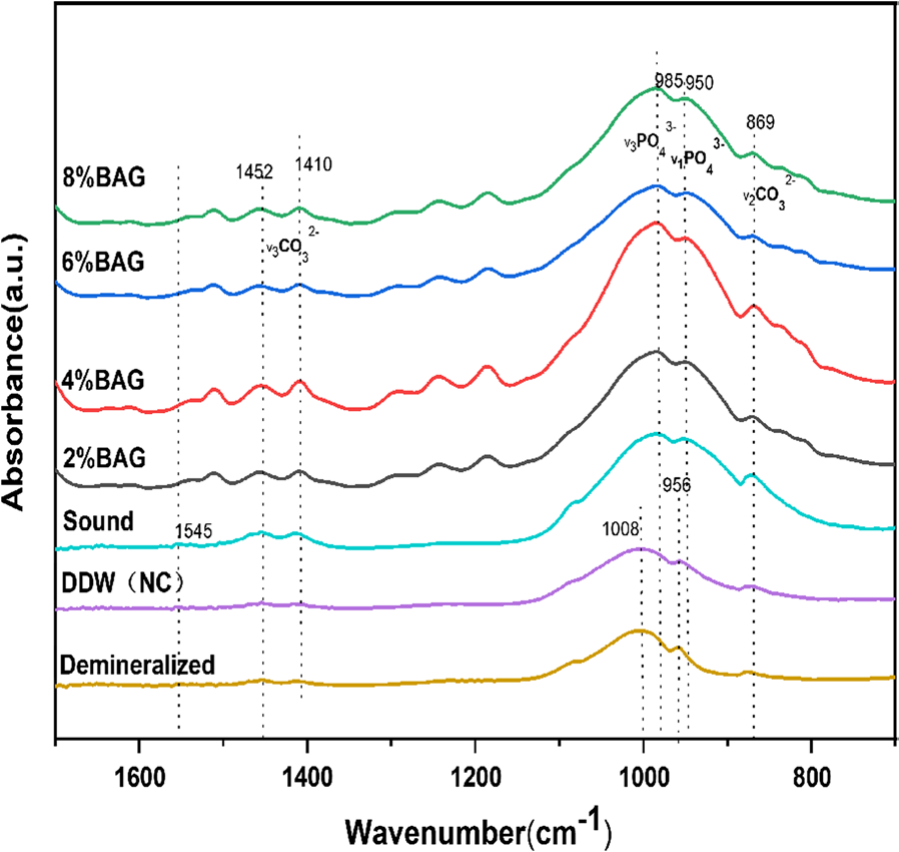
FT-IR/ATR spectra of each group

The representative FT-IR/ATR spectrum of each group is shown in Fig. 4. In sound enamel, the strongest bands were observed at 985 cm^–1^ and 950 cm^–1^, which may be attributed to the v_3_PO_4_^3−^ and the v_1_PO_4_^3−^ vibration respectively [17]. The two peaks were shifted to higher wavenumbers (1008 cm^−1^ and 956 cm^–1^) after demineralization and shifted back after pH cycling in all the experimental groups except for the DDW group. A band at 869 cm^–1^ was found in all the groups which is attributed to v_2_ CO_3_^2–^ vibration [17], and the major component of it is type B carbonate (β-HCA) [18]. v_3_ CO_3_^2–^ of β-HCA presented bands at 1410 cm^−1^ and 1452 cm^−1^. These two bands showed relatively higher intensity in the BAG group. No band at 880 cm^−1^ and 1545 cm^−1^ was seen in BAG groups, indicating that no type-A HCA was found.

## Discussion

A chemical cariogenic model was used in our study instead of a complex bacterial biofilm model. In fact, most in vitro studies on cariology used simple chemical models to generate artificial carious lesions [19]. It has obvious advantages such as simplicity of a study, cost and time saving, controllable experimental conditions, as well as reproducibility of the experiment. Recent studies showed that carious lesions created by chemical models exhibited several characteristics similar to natural caries regards to mineral loss and mechanical property [20]. Hence, these lesions were acceptable in cariology research to create artificial enamel lesions.

Alteration of the chemical composition in demineralized enamel is usually accompanied by changes in mechanical properties [21]. Microhardness testing has been used to provide information on such physical property and to quantify alterations in dental tissue in response to de-and remineralization protocols [22, 23]. Deciduous tooth enamel has lower inorganic content than permanent tooth; thus, it has a lower microhardness. The mean baseline micro-hardness values of deciduous enamel samples ranged from 340.45 ± 15.62 to 345.84 ± 6.97 VHN in our study, which was in accordance with that of Molla Asadollah et al. [24] Surface hardness increased after pH-cycling in all groups indicating mineral gain happened. BAG exhibited superior recovery capacity in accordance with other studies [25] since the lowest concentration of it successfully restored the surface hardness, and showed significantly higher % REMH than the DDW. The 6% BAG displayed the best performance of % REMH, reaching 57.40% ± 1.72, followed by 4% BAG and 8% BAG, suggesting that re-harden capacity is not concentration-dependent. Excess Ca and P ions may limit the remineralization progress when reaching the ions saturation. Interestingly, DDW also rehardened the demineralized tooth to some extent, which may be due to the remineralization solution it was immersed in, offering Ca and P ions to the demineralized enamel, although not much [22].

Morphology maybe another feature of the mineral change [21]. After pH-cycling,BAG groups exhibited surface morphology with micro-spheres covered the entire lesion surface, which could be explained by its mineralizing traits to form a calcium-phosphate complex by chemical bonds [9]. In addition, acting as a biomimetic mineralizer [9], the facial surface of a growing apatite could become a nucleating site for another, thus stacked layers were formed instead of a single one in BAG groups. Among them, the 6% BAG group presented the most uniform and dense surface, precipitation of apatite-like crystals repairing almost whole surface defects of artificial caries. Interestingly, evenly distributed precipitation was also found in the DDW (e) group probably due to the artificial saliva it immersed in. The thin and single layer suggested that it failed to function as a nucleator of calcium phosphate to form a dense stack surface.

To quantitatively evaluate mineral changes on the enamel surface, the mass fraction of the calcium and phosphorus ions were determined. It was found that Ca(wt%) and P(wt%) decreased after demineralization indicating rapid mineral loss while increased after remineralization in all groups suggesting mineral gain. BAG groups showed more mineral gains than the DDW, and saturation of calcium and phosphate ions reached at 6% concentration. The main component of tooth enamel is hydroxyapatite, but there may also be impurities (such as carbonate), which could reduce the Ca/P to a certain extent [18, 26]. The mean Ca/P value of sound deciduous teeth in this experiment was 1.60, which is lower than the theoretical value of 1.67 [17]. No significant difference was found in the Ca/P ratio between different BAG groups, which can be proposed that concentration is not a direct factor in changing the chemical composition of the deposition. Generally, the higher the Ca/P ratio, the lower the precipitate solubility [15]. The ratio was lower in BAG groups than sound teeth, probably owing to the co-precipitation of calcium carbonate and silicon phosphate [27]. Thus it can be speculated that the co-precipitations formed by BAG exert poorer acid resistance than normal deciduous teeth.

In order to identify the calcium phosphate complexes of the newly formed layer, FT-IR/ATR was carried out in each group. After demineralization, the peak of v_3_PO_4_^3−^ and v_1_PO_4_^3−^ shifted to higher wavenumbers, indicating that the P–O bond length was reduced [21]. The framework of the apatite comprises P–O–Ca atomic bridges, thus a shortening of P–O bonds suggests lengthening of the adjacent Ca–O bonds, which increases the release of Ca from enamel [21]. In contrast, after pH-cycling, the v_3_PO_4_^3−^ and v_1_PO_4_^3−^ vibration bands in all experimental groups had shift to lower wavenumbers except for the DDW group, indicating strengthened Ca–O bonds. In addition, the substitution of carbonate in apatite is most readily detected by FTIR [28], and our study showed that the newly formed layer of BAG groups is composed of β-HCA. The carbonate ion is known to occupy two different positions in the hydroxyapatite of the enamel, the hydroxide position (A) and the phosphate position (B) [28, 29] and both will cause distortions in the hydroxyapatite structure. The carbonate ion in the hydroxide position will distort the lattice more than in the phosphate position thus the carbonate ion will be less tightly bound in the A position than in the B position [28]. Besides,B-type carbonate is considered to dominate the apatitic formation of biominerals in physiological conditions [29] rather than A-type.

This is the first study to evaluate the exact efficacy of the independent application of 45S5 BAG for remineralization on deciduous enamel. It seems that 6% may be the optimal concentration for children, considering its performance on the best microhardness recovery and the most ions deposition. However, the results reported should be taken with caution, because EDX is semi-quantitative [30] and area-specific analysis for element analysis since the newly forming layer was not evenly distributed. Future studies should focus on more reliable quantitative methods and assess its abrasion durability, which is a key requisite for survival in the oral cavity.

## Conclusion

The study gives targeting evidence on applying 45S5 BAG for children's health intervention since it successfully recovered enamel surface mechanical property, morphology and chemical elements. Among them, 6% BAG exhibited the greatest overall efficiency. Considering the potential risk of toxicity and fluorosis with fluoride use, 45S5 BAG, a new biocompatible material which exerted excellent remineralization performance, may have a promising future use for advanced caries management in children.

## Data Availability

The datasets used and/or analysed during the current study available from the corresponding author on reasonable request.

## Abbreviations

BAG: Bioactive glass
DDW: Deionized water
REMH: Recovery of enamel microhardness
SEM: Scanning electron microscopy
EDS: Energy-dispersive X-ray spectroscopy
FT-IR/ATR: Fourier transform infrared spectroscopy equipped with attenuated total reflectance
HCA: Hydroxycarbonate apatite
